# Synthesis and Biological Properties of Caffeic Acid-PNA Dimers Containing Guanine

**DOI:** 10.3390/molecules18089147

**Published:** 2013-07-31

**Authors:** Maria Gaglione, Gaetano Malgieri, Severina Pacifico, Valeria Severino, Brigida D’Abrosca, Luigi Russo, Antonio Fiorentino, Anna Messere

**Affiliations:** Department of Environmental, Biological and Pharmaceutical Sciences and Technologies, Second University of Studies of Naples, Via Vivaldi, 43, 81100 Caserta, Italy; E-Mails: maria.gaglione@unina2.it (M.G.); gaetano.malgieri@unina2.it (G.M.); severina.pacifico@unina2.it (S.P.); valeria.severino@unina2.it (V.S.); brigida.dabrosca@unina2.it (B.D.); luigi.russo2@unina2.it (L.R.); antonio.fiorentino@unina2.it (A.F.)

**Keywords:** caffeic acid, PNAs, caffeic acid-amide derivatives, biological properties, cytoprotective activity

## Abstract

Caffeic acid (CA; 3,4-dihydroxycinnamic acid) is endowed with high antioxidant activity. CA derivatives (such as amides) have gained a lot of attention due to their antioxidative, antitumor and antimicrobial properties as well as stable characteristics. Caffeoyl-peptide derivatives showed different antioxidant activity depending on the type and the sequence of amino acid used. For these reasons, we decided to combine CA with Peptide Nucleic Acid (PNA) to test whether the new PNA-CA amide derivatives would result in an improvement or gain of CA’s biological (*i.e*., antioxidant, cytotoxic, cytoprotective) properties. We performed the synthesis and characterization of seven dimer conjugates with various combinations of nucleic acid bases and focused NMR studies on the model compound *ga*-CA dimer. We demonstrate that PNA dimers containing guanine conjugated to CA exhibited different biological activities depending on composition and sequence of the nucleobases. The dimer *ag*-CA protected HepG2, SK-B-NE(2), and C6 cells from a cytotoxic dose of hydrogen peroxide (H_2_O_2_).

## 1. Introduction

Hydroxycinnamic acids (HCA) and derivatives are well-known phenolic compounds ubiquitous in plants, showing relevant antioxidant properties as well as cytotoxicity toward several tumor cell lines. They are able to inhibit cell growth in a manner strongly dependent on their structural properties [1,2]. Caffeic acid (CA; 3,4-dihydroxycinnamic acid) which is a member of the HCA family is endowed with a high antioxidant activity. This property is due to the delocalization of an unpaired electron caused by the extended conjugated side chain. In addition, the *ortho*-dihydroxyl group of CA forms a hydrogen bond, which creates a more stable configuration after breaking the O–H bond [3]. In recent years, HCA derivatives have gained a lot of attention due to their antioxidative [4], antitumor [5], antimicrobial [6,7] and cytoprotective [8,9,10,11] properties. Owing to the biological properties of caffeic acid analogues, several research groups have started to explore strategies for their synthesis in order to obtain compounds with improved biological activities. Many CA-derived ester analogues (e.g., CAPE) have been shown to protect neurons against oxidative damage induced by H_2_O_2_ or by other oxidative stress conditions [12]. However their use is limited as they are metabolically very labile due to a rapid decomposition by esterases that leads to their low bioavailability *in vivo* [13]. Several modified CA amides (CAPA) have recently shown more stable characteristics and an anti-lipooxidation activity [14]. A series of catechol ring-fluorinated derivatives of CA phenethyl amide were synthesized and screened for cytoprotective activity against H_2_O_2_ induced oxidative stress [15]. In addition, caffeoyl-peptide derivatives have been reported to have enhanced antioxidant activity, showing different performances depending on the type and the sequence of amino acid used [16,17,18,19,20]. Peptide Nucleic Acid (PNAs) are bioactive DNA analogue constituted of *N*-(2-aminoethyl)glycine subunits forming structurally uncharged and achiral polyamide backbones in which nucleobases are inserted on *N*-glycine by methylene carbonyl linkage [21]. Important features of PNAs are the strengthened binding-affinity to complementary nucleic acids and, more interestingly, the high biological and chemical stability [21]. Since its discovery, PNA has attracted major attention in the chemistry and biology fields because of its interesting chemical, physical, and biological properties. PNA is chemically stable and resistant to enzymatic cleavage and thus expected to be no degraded in cells. PNA synthesis employs protocols derived from solid phase peptide synthesis resulting in a straightforward and flexible methodology. PNAs are involved in expanding set of applications, including pharmaceutical and diagnostic applications as genome mapping, identification of mutations and measurement of telomere length [22]. Cellular uptake, cellular localization and specific targeting of PNAs are largely discussed in applications in which PNAs act as oligonucleotide analogs, in antisense/antigene experiments as well as diagnostic tools. The cellular uptake of polyPNA is very slow, and is still considered to be the major challenge to overcome before it can be used as a therapeutic drug. Laser confocal microscopy studies revealed a variable uptake pattern of PNA oligomers ranging from clear endosomal localization to a somewhat diffuse cytoplasmatic and sometimes nuclear localization depending on the specific combination of PNA sequence and cell type was observed. This unique nucleic acid analog specifically recognizes DNA or RNA fragments and forms duplexes or triplexes *via* Watson-Crick and Hoogsteen base pairing. The affinity of PNA for DNA or RNA is higher than that of the natural oligonucleotides and of other analogs, both because of the loss of negative charges and the properties of the polyamide backbone [23]. Recent attempts to provide further applications of this exciting nucleic acid analog propose modifications of backbone and the development of novel base analogs. Here, we propose the inedited conjugation of PNA to caffeic acid. The combination of unnatural molecules as PNAs with natural molecules as Caffeic Acid could be a novel tool to enhance the biological value of this cinnamic acid derivative: the electrically neutral nature of PNAs could facilitate the cellular delivery of the PNA-caffeic acid conjugates. Furthermore, the PNA heterocyclic bases could assist the caffeic acid in its biological performances depending on the nucleobase nature and sequence. In particular, we have focused our attention on the PNA-CA dimers containing guanine (CA-*xg*-NH_2_ and CA-*gx*-NH_2_, CA is at the N-terminus of PNA dimer), where *x* is either adenine or cytosine or thymine or guanine nucleobase. The Guanine nucleobase was selected for its hydrogen bond accepting and donating groups and for its well-known lowest ionization energy among the four nucleobases, especially when flanked by purine nucleobases [24]. Here, we report the synthesis and the chemical characterization of the PNA-CA conjugate dimers **1**–**7** (Table 1) together with the evaluation of their antioxidant and cytotoxic activities. The most interesting *ag*-CA dimer was also evaluated for cytoprotective effect.

**Table 1:** Crude yield and MALDI-TOF MS analysis of CA-PNA dimers.

Compounds	Crude yield (%)	Theoretical molecular mass	Experimental molecular mass [M+H]^+^	Δ (Da)
CA-*ag*-NH_2_ (**1**)	45	745.27	746.28	0.01
CA-*ga*-NH_2_ (**2**)	50	745.27	746.28	0.01
CA-*cg*-NH_2_ (**3**)	55	721.27	722.27	0.00
CA-*gc*-NH_2_ (**4**)	50	721.27	722.27	0.00
CA-*tg*-NH_2_ (**5**)	45	736.27	737.27	0.00
CA-*gt*-NH_2_ (**6**)	55	736.27	737.27	0.00
CA-*gg*-NH_2_ (**7**)	40	761.27	762.27	0.00

## 2. Results and Discussion

### 2.1. Synthesis of Dimers ***1***–***7***

**Scheme 1:**
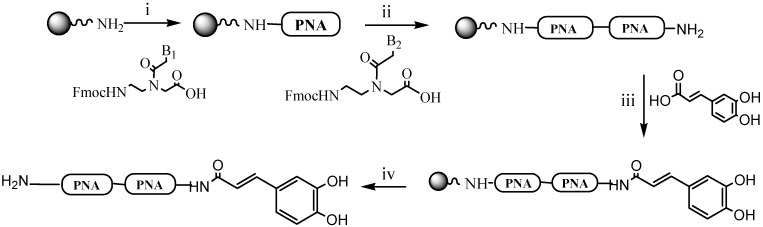
Solid-phase synthesis of CA-PNA dimers. B_1_= guanine and B_2_= adenine in 1; B_1_= adenine and B_2_ = guanine in 2; B_1_ = guanine and B_2_ = cytosine in 3 B_1_ = cytosine and B_2_ = guanine in 4; B_1_ = guanine and B_2_ = thymine in 5; B_1_ = thymine and B_2_ = guanine in 6; B_1_ and B_2_ = guanine in 7.

We have synthesized PNA-CA dimers containing guanine (CA-*xg*- NH_2_ and CA-*gx*-NH_2_), where *x* is either adenine or cytosine or thymine or guanine nucleobase. The conjugates **1**–**7** were synthesized exploiting the well known strategy developed for the solid phase synthesis of PNAs [21], using Fmoc protection of the backbone amino function and Bhoc protection for the exocyclic amino function of the nucleobases. The coupling activation was performed with reagents known from peptide synthesis. In particular, HATU was used for the PNA monomers and PyBOP for caffeic acid couplings. This last activation was chosen because it yields a better coupling for caffeic acid compared to that obtained using HATU activation. CA-PNA dimers were obtained in good yields (Table 1) by solid-phase peptide synthesis on Rink amide (aminomethyl)polystyrene resin (Scheme 1). The dimers were prepared on Rink amide (aminomethyl)polystyrene resin (~1.1 mmol/g loading). Each reaction step was monitored by UV-Fmoc test, measuring the UV absorbance of the weighted sample after deprotection using 20% piperidine in DMF.

*Reagents and conditions*: i, ii 0.2 M HATU in DMF, 0.8 M monomer in base solution (Applied Biosystems) 1 h at R.T.; iii CA (2 equiv.), HOBT(2 equiv.), PyBOP (2 equiv.) and DIEA (4 equiv.) in dry DMF, over night at R.T.; iv TFA/m-cresol (4:1, v:v), 2 h.

In order to confirm the identity of compounds, the crudes were analysed by MALDI-TOF MS in reflectron positive ion mode [25] (Table 1).

The different physico-chemical properties of dimers **1**–**7** were predicted by theorical calculations and compared to CA. The values of the partition coefficient (*C*Log P) and of the aqueous solubility (*C*Log S) of PNA-CA dimers **1**–**7** were obtained using the ChemDraw Ultra 10.0 (CambridgeSoft) [26] and ALOGPS 2.1 programs [27] respectively and compared to those of CA (Table 2). In particular, a decrease of *C*Log P was observed for all the PNA-CA conjugates. This would indicate a lower lipophilicity of the CA when conjugated to PNA. On the other hand, the values of the *C*Log S increase in the conjugates CG-CA, GC-CA, GG-CA, GT-CA, TG-CA. For the conjugates **1** and **2**, the *C*Log S value is lower than that of CA indicating that these two dimers would be more soluble in water compared to the other conjugates. For these reasons, PNA-CA dimers **1** and **2** might have a potential good bioavailability as they include both hydrophobic and hydrophilic parts in one molecule [28,29].

**Table 2:** Partition coefficient (*C*Log P) and aqueous solubility (*C*log S) of CA-PNA dimers.

Compounds	*C*Log P ^a^	*C*Log S ^b^
Caffeic acid (CA)	0.97	−1.41
CA-*ag*-NH_2_ (**1**)	−2.24	−1.73
CA-*ga*-NH_2_ (**2**)	−2.24	−1.73
CA-*cg*-NH_2_ (**3**)	−2.52	−1.12
CA-*gc*-NH_2_ (**4**)	−2.52	−1.12
CA-*tg*-NH_2_ (**5**)	−2.0	−1.10
CA-*gt*-NH_2_ (**6**)	−2.0	−1.10
CA-*gg*-NH_2_ (**7**)	−3.23	−1.12

^a^ Calculated by ChemDraw Ultra 10.0 (CambridgeSoft) and ^b^ ALOGPS 2.1 program.

### 2.2. Chemical Characterization of Dimer ***1***

**Figure 1:**
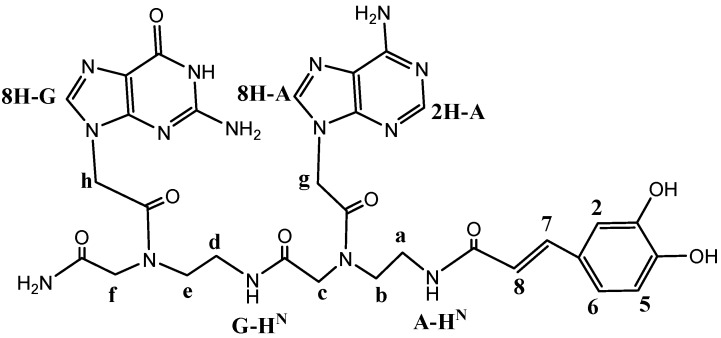
Atom numbering system used in NMR spectral analysis for dimer **1**.

In order to provide a complete physico-chemical characterization of the new synthesized PNA-CA dimers, dimer **1** was chosen as model compound. It was analyzed *via* elemental analysis, NMR and HPLC (the atom numbering system used in NMR spectral analysis is given in Figure 1).

**Figure 2:**
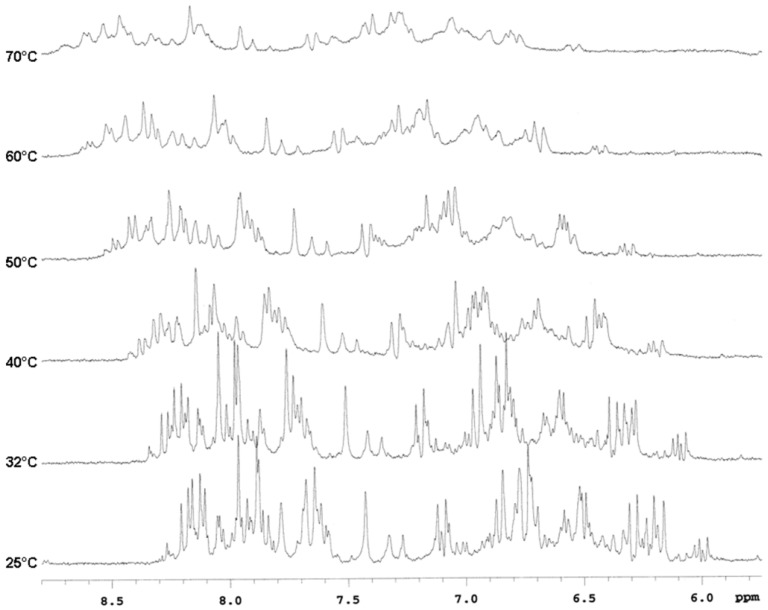
^1^H-NMR spectra for derivative **1** acquired from 25 to 70 °C.

The elemental analysis (calculated C, 49.93; H, 4.73; N, 28.17, found C, 50.36; H 4.77; N 28.23) established the molecular formula of the dimer **1** to be C_31_H_35_N_15_O_8_. Accordingly, a 2D-DOSY experiment with a D_trans_ value of (2.9 ± 0.2) × 10^−10^ m^2^ s^−1^ at R.T. was consistent with a monomeric state for **1**. The ^1^H-NMR spectra for dimer **1** were quite complex. In particular, the aromatic region showed more than one set of signals for each type of proton ascribable to nucleobase and CA residues consistent with the existence of several distinct, but structurally related molecules, or with the presence of a mixture of two or more conformers in slow exchange. As largely reported in literature, in solution the PNA monomer exists as both the *cis* and *trans* rotamers about the tertiary amide bond, slightly favoring the *trans* conformation as revealed from the ^1^H-NMR spectra. The two rotamers interconvert at room temperature. For longer sequences, the ^1^H-NMR spectrum reveals the existence of a multitude of structural species. For a total of N number of residues, there can be 2^N^ possible structural species. Variable temperature NMR experiments are usually employed to follow the interconversion of the rotamers in detail by analyzing the patterns of coalescence between various pairs of signals. This latter hypothesis was confirmed by the comparison of the ^1^H-NMR experiments acquired at rising temperatures from 25 to 70 °C (Figure 2). It was possible to observe a significant broadening or a change in the chemical shift of some diagnostic signals in the aromatic region: at 32 °C some chemical shifts were already changing as the rate of equilibration in the rotamer mixture increased. This confirms that dimer 1 existed in solution as mixture of rotamers resulting from hindered rotation of the amide C–N bonds.

In the downfield region of ^1^H-NMR spectrum of compound **1**, two olefinic protons appearing as coupled doublets (15.9 Hz) at δ 7.11 and 6.28 and three aromatic protons of a 1,2,4-trisubstituted benzene ring at δ 6.84, 6.77 and 6.73 were evident, which confirmed the presence of a CA residue. Diagnostic signals of H-2 and H-8 protons relative to adenine and H-8 proton of guanine were observed at 8.48, 8.20 and 8.17 ppm respectively. Similar ^1^H-NMR spectra were collected for derivative **2**.

The identity of the derivative **1** was further confirmed using 2D spectra such as TOCSY, ROESY and ^1^H-^13^C HSQC. Because of the well-known restricted rotation around the tertiary amide bond in PNA that produces two possible rotamers in solution around the tertiary amide bond, the spin systems in the TOCSY and ROESY spectra were multiplied [30]. The backbone region of the 2D-TOCSY recorded in water showed, among others with minor intensity, four main spin systems regarding the two amidic protons coupled with methylene protons *a*, *b* and *d*, *e* (H^N^ at δ 8.51 coupled with δ 3.78 and 3.40; H^N^ at δ 8.45 coupled with δ 3.47 and 3.12; H^N^ at δ 8.20 coupled with δ 3.66 and 3.55; H^N^ at δ 8.09 coupled with δ 3.44 and 3.22). A NOE cross-correlation peak between the adenine H-8 and methylene protons *g* (δ 8.20 and 5.09 respectively) and between the guanine H-8 and methylene protons *h* (δ 8.17 and 5.17 respectively) were clearly identified in the 2D-ROESY experiment in D_2_O confirming the presence of the two nucleobases. Moreover, NOE cross-correlation peaks between the two olefinic protons and the 1,2,4-trisubstituted benzene ring protons were also found. The olefinic proton H-7 (δ 7.11) showed a clear NOE with H-2 (δ 6.84) while the proton H-8 (δ 6.28) showed two clear NOE with H-5 and H-6 (δ 6.73 and 6.77 respectively). The ^13^C chemical shifts of the carbons are consistent with the proton assignments made (*i.e.*, the diagnostic olefinic signals in the ^1^H-^13^C HSQC were at δ 7.11\143.60 and 6.28\120.40).

**Figure 3:**
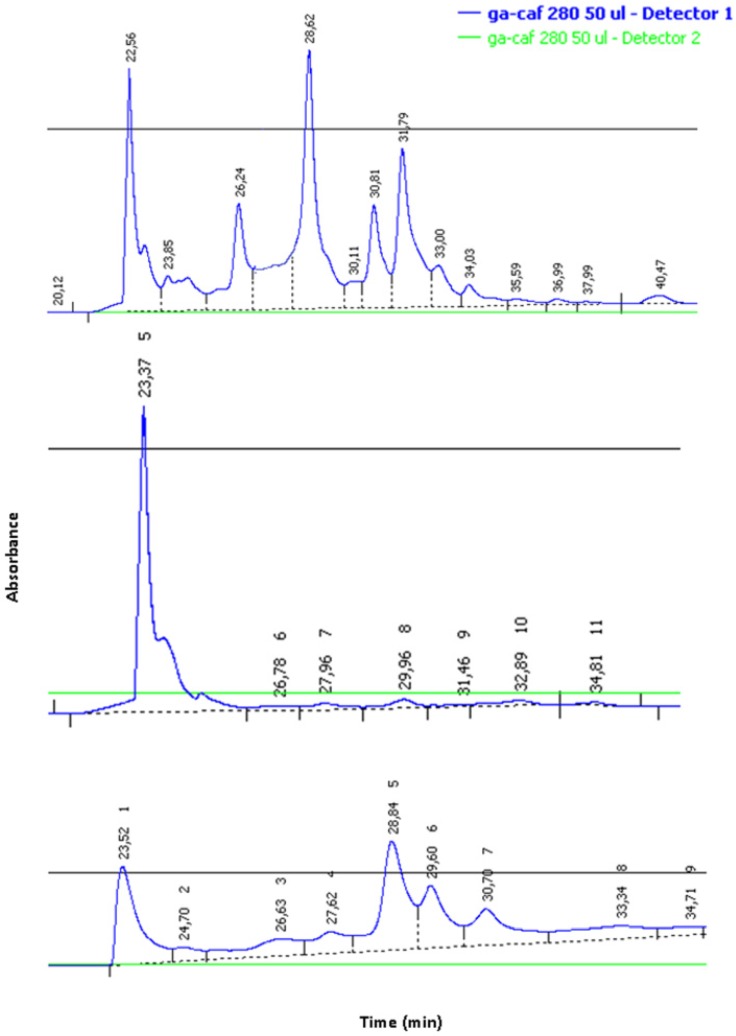
HPLC profile of **1**. Crude mixture (**a**), isolated conformer (**b**), re-established mixture of rotamers (**c**).

Moreover, the different rotamers were also separated by reverse phase HPLC and resulted stable as solids but, when dissolved in H_2_O, they interconverted, re-establishing the original mixture in *ca*. 24 h (Figure 3).

### 2.3. Biological Characterization

**Figure 4:**
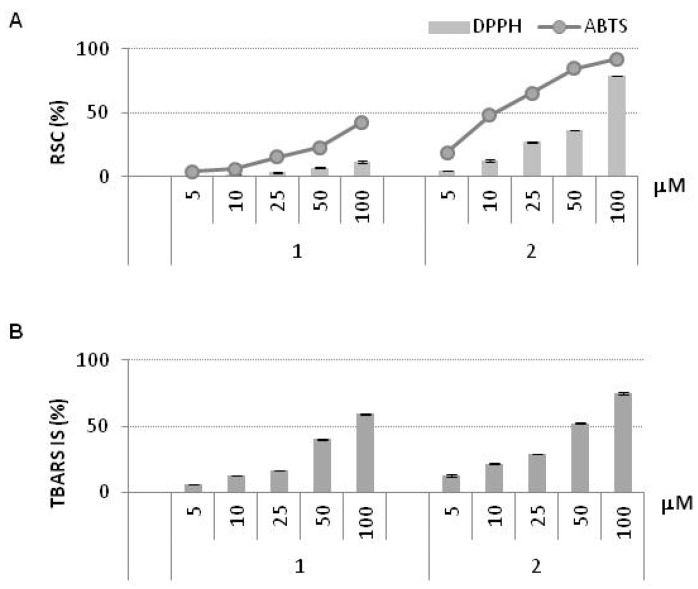
Antioxidant activity of PNA-CA dimers 1 and 2. (**A**) Radical Scavenging Capacity (RSC, %) towards DPPH^•^ and ABTS^•+^ (**B**) TBARS Inhibition Synthesis (TBARS IS, %). Values, reported as percentage *vs*. a blank, are the mean value ± SD of measurements carried out on 3 samples (n = 3) analyzed three times.

The antioxidant properties of CA and of PNA-CA dimers **1**–**7** were evaluated by using different *in vitro* antioxidant assays such as 2,2′-azino-bis(3-ethylbenzothiazoline-6-sulfonic acid) radical ABTS [31], 2,2-diphenyl-1-picrylhydrazyl (DPPH) free radical scavenging and by using a method for estimating lipid peroxidation, through the ability to react with thiobarbituric acid (TBA) that yields “thiobarbituric acid reactive substances” (TBARS method) [32]. The free radical scavenging capacities (RSC) of various concentrations of CA (2.5–100.0 μM) and PNA-CA dimers **1**–**7** (5.0–100.0 μM) were evaluated. No significant antioxidant and free radical scavenging activities were registered for derivatives **3**–**7** (data no shown) while dimers **1** and **2** gave significant results (Figure 4A). Although with a lower activity compared to CA, the dimer **2** was effective in scavenging the radical target species exhibiting IC_50_ = 51.36 µM towards DPPH radical and 13.91 µM towards ABTS radical (Table 3). The antiradical capability was strongly dependent on the dose tested (Figure 4A); at 100 µM, both radical species were almost completely converted in their reduced forms. In particular, the ABTS cation radical was reduced by 92.0% and DPPH radical was reduced by 78.1%. On the other hand, the dimer **1** showed low activity towards the selected radical species in the whole dose range tested. When the TBARS method was applied, a similar trend was observed (Figure 4B). The derivative **2** was more effective than **1** to prevent the formation of thiobarbituric reactive species. In fact, the dose required to reach a 50% inhibition (IC_50_) was 41.38 µM while for **1** was 85.34 µM (Table 3).

**Table 3:** DPPH, ABTS, and TBARS IC_50_ (μM) values of PNA-CA dimers **1** and **2**. IC_50_ values, defined as the compound amount causing 50 per cent activity inhibition, was determined from the plotted curves.

*Compounds*	*DPPH IC_50_*	*ABTS IC_50_*	*TBARS IC_50_*
CA-*ag*-NH_2_ (1)	>100	>100	85.34
CA-*ga*-NH_2_ (2)	51.36	13.91	41.38

Dimer **2** exhibited higher activity than **1** in both the free radical scavenging test and the lipid peroxidation inhibition test despite the same hydrophilic properties. The DPPH radical scavenging assay was carried out in methanol, which is a hydrophilic solvent, whereas the lipid peroxidation inhibitory assay was performed in Tween 40-emulsified rapeseed oil. Table 1 shows that PNA-CA dimers **1** and **2** had identical *C*Log P (−2.24) and *C*Log S (−1.73). We expected the same accessibility to the lipid emulsion for **1** and **2** CA-PNA dimers as they contained the same hydrophobic and hydrophilic moieties and the same solubility in polar solvents. The different effectiveness of the two investigated CA conjugates suggested that the observed antioxidant activities were strongly dependent on the sequence of the PNA nucleobases in the dimers and in particular on the PNA monomer close to CA. In fact, in the dimer **2** the guanine ring provides additional stability to CA hydroxyl radical after the free radicals are quenched, whereas dimer **1**, because of its different structure, does not benefit of the same effect. The electrophilic OH· radical adds to the C(2), C(4), C(5) and C(8) positions on the guanine base, as well as on the adenine. The purine base guanine has the highest affinity for reaction with the OH radical, as can be inferred from its reaction rate constant [33,34]. The relative reactivity of the CA in the dimers was influenced by the surrounding bases. In the dimer **2**, CA was flanked by guanine base which could accept OH· radical of CA more efficiently than adenine base, determining the better antioxidative activity of **2**.

Effects of PNA-CA dimers on cell proliferation and on protection from oxidative stress caused by hydrogen peroxide (H_2_O_2_) in the three different cell lines were also evaluated by the 3-(4,5-dimethyl thiazol-2-yl)-2,5-diphenyl-tetrazolium bromide (MTT) assay [32].

**Figure 5:**
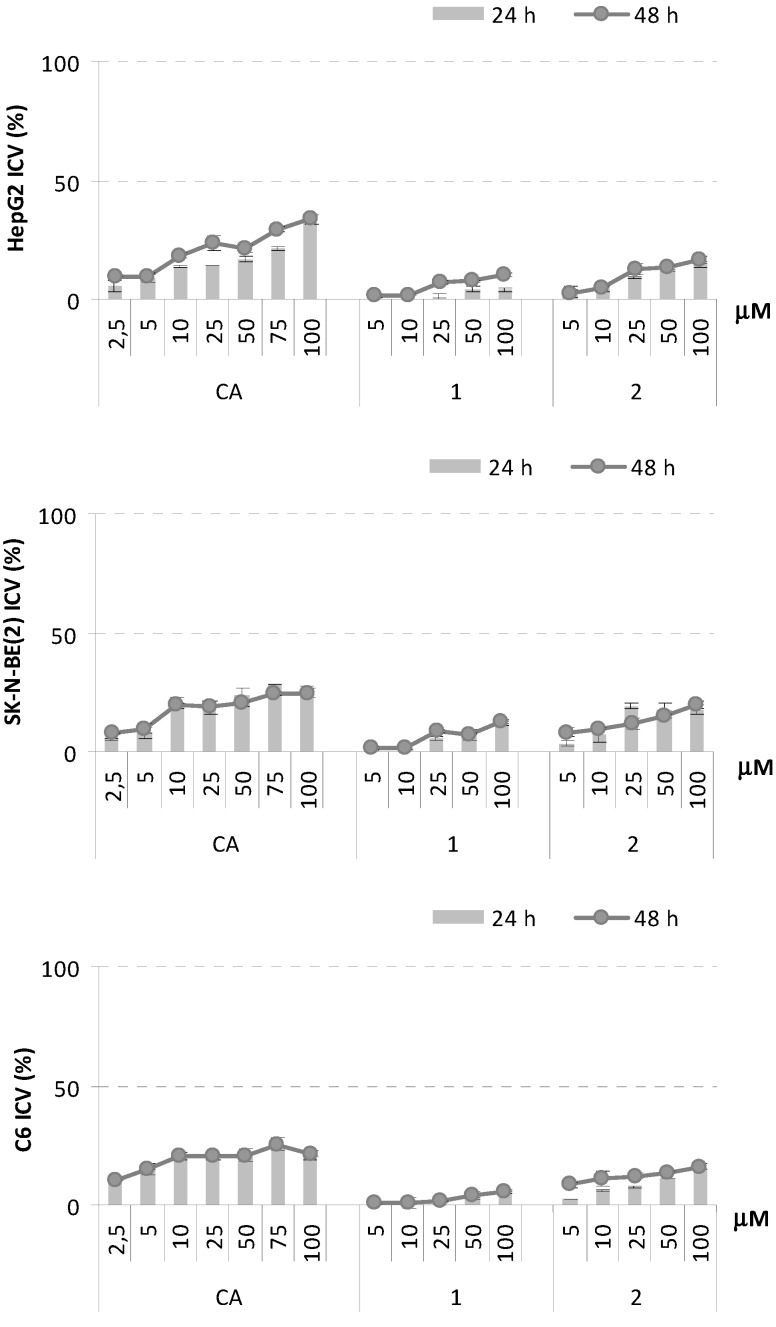
Inhibition of Cell Viability (ICV, %) of CA, PNA-CA dimers **1** and **2** on HepG2, SK-B-NE(2), and C6 cell lines. Values, reported as percentage *vs*. an untreated control, are the mean value ± SD of measurements carried out on three samples (n = 3) analyzed twelve times.

The choice of the tested cell lines was based on literature reports. HepG2 (human hepatocellular carcinoma) cell line is commonly used as a model system for detecting cytoprotective agents [35,36]. Analogously, rat C6 glioma cells (Rat Glioma cell line) and SK-N-BE(2) (human neuroblastoma cell line) neuroblastoma ones were cultured in the presence of cytoprotective natural products to determine their protective effect against H_2_O_2_-induced cytotoxicity [37,38]. It was also previously showed that glial cells are more resistant than neurons to oxidative stress induced by application of H_2_O_2_ and these cells are thought to play an important role in antioxidant defense mechanisms [39]. HepG2, SK-N-BE(2) and C6 were treated with five increasing concentrations of CA and conjugates **1**–**7** (5.0–100.0 µM), at two different exposure times (24 and 48 h). The dimers exerted only a mild inhibition of cell growth of all the tested cell lines. In particular, when the three cell lines were treated with compound **2**, a cell viability dose-dependent inhibition was observed; exposure time did not affect antiproliferative response. Indeed, compound **1** showed a different effect on the proliferation of the three tested cell lines: SK-N-BE(2) cell line appeared to be more responsive than HepG2 and C6 cells (Figure 5).

**Figure 6:**
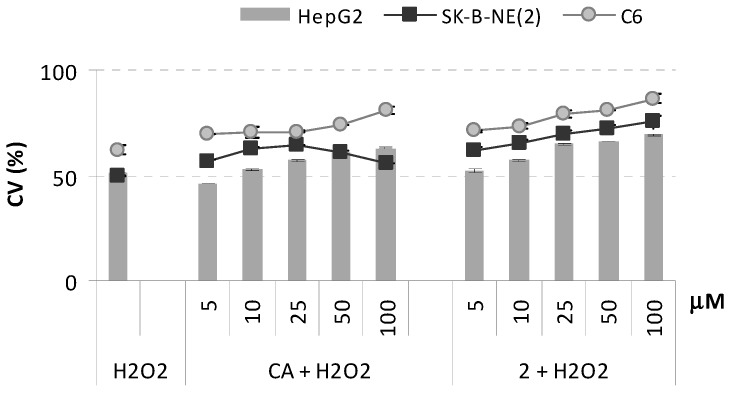
Cytoprotective effects of CA, and PNA-CA dimer **2** against H_2_O_2_ (500 µM) induced oxidative stress on HepG2, SK-B-NE(2), and C6 cell lines. Values of Cell Viability (CV, %), reported as percentage *vs*. an untreated control, are the mean value ± SD of measurements carried out on 3 samples (n = 3) analyzed twelve times.

The weak cytotoxic effects recorded for derivative **2** led us to hypothesize that this molecule, just like other natural and synthetic caffeic acid derivatives, could play a role in cytoprotection mechanisms. Oxidative stress is involved in various neurodegenerative diseases, stroke and ischemia/reperfusion injury. It is well established that treatment of cells with H_2_O_2_ results in nuclear damage, decrease in mitochondrial transmembrane potential, cytosolic accumulation of cytochrome-c, activation of caspase-3, increase in the formation of reactive oxygen species. Protection of cells against H_2_O_2_-induced cell death can therefore be used to measure the cytoprotective activity of test agents. Here, the cytoprotective effects of dimer **2** on hydrogen peroxide (H_2_O_2_)-induced apoptosis in HepG2, SK-B-NE(2), and C6 cell lines were investigated. As shown in Figure 6, the dimer exhibited slight but statistically significant improved cytoprotective activity compared to CA, specially on SK-N-BE(2) cell line. In particular, it was observed that, in all the three tested cell lines, the molecule was responsible for a dose-dependent protection against H_2_O_2_-mediated cytotoxicity.

### 2.4. *In Silico* Conformational Study

**Figure 7:**
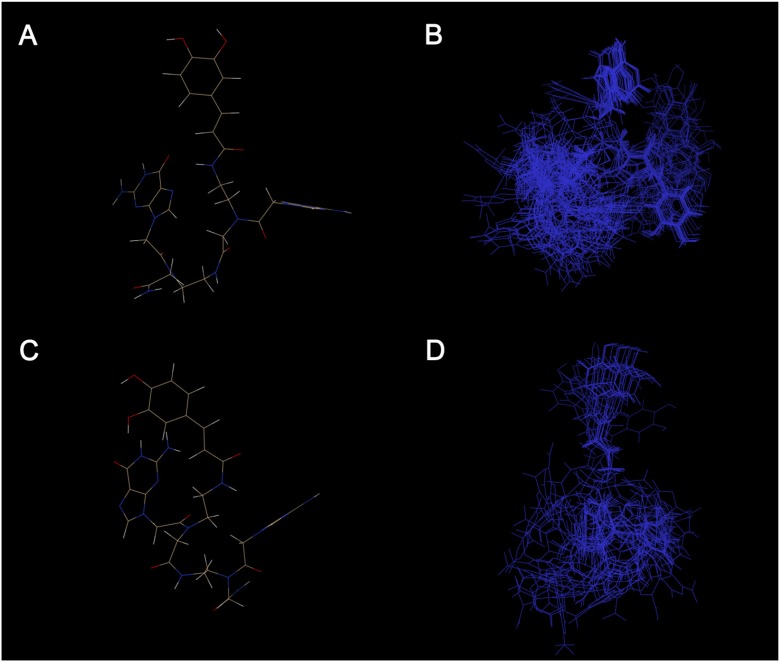
The proposed molecular structure of the compound **1** (**A**) and compound **2** (**C**). Structure ensemble of the conformations generated and selected by Cyndi for compound **1** (**B**) and compound **2** (**D**).

In order to have further information on the dimers **1** and **2** that may be related to the differences in bioactivity, we explored the conformational space sampled by the two compounds. In fact, molecules containing flexible structural elements should be represented by ensembles of physically relevant conformers, interchanging over the time. For these reasons we have used the software Cyndi [40] to characterize the conformational ensemble for derivatives **1** and **2** in order to evaluate their molecular flexibility. Our results, according to the NMR data reported, indicate that the two molecules exist in multiple different conformers. In particular, compound **1** (Figure 7C,D) showing more intrinsic flexibility than compound **2** (Figure 7A,B) can populate a wider conformational space. The higher bioactivity showed by compound **2** is likely also explained by the smaller conformational space sampled by it.

## 3. Experimental

### 3.1. Synthetic Procedure for CA-PNA Dimers

The dimers were prepared by conventional Fmoc solid-phase peptide synthesis on Rink amide (aminomethyl)polystyrene resin (~1.1 mmol/g loading). Each reaction step was monitored by UV-Fmoc test, measuring the UV absorbance of the weighted sample after deprotection using 20% piperidine in DMF*.* The carboxylic acid group of the protected monomer (0.215 M in dry DMF) was activated by mixing PNA activator (0.2 M HATU in DMF) and monomer in 0.8 M base solution (Applied Biosystems) for at least 2 minutes and then added to the resin. Typically, a 5× stoichiometric excess was used for all monomers. After 1 h at R.T., the resin was washed with DMF, 5% DIEA in DCM and finally with DCM. The capping reaction was done by adding acetic anhydride to the resin, resulting in conversion of all unreacted sites to the acetamides. After the synthesis and deprotection of the PNA dimer, CA (2 equiv.), HOBT (2 equiv.), PyBOP (2 equiv.) and DIEA (4 equiv.) in dry DMF were added and the coupling reaction was performed over night at R.T. CA-PNA dimers were detached from the resin by treating with a solution TFA/*m*-cresol (4:1, v:v) for 2 h. The procedure also removed the Bhoc protecting group from the nucleobases. The crude was filtered and the solution was concentrated in high vacuum, then triturated with cold diethyl ether. The characterization of all conjugates was accomplished via MALDI-MS Spectrometry (MALDI-TOF micro MX, Waters Co., Manchester, UK, equipped with a pulsed nitrogen laser, λ = 337 nm). In order to confirm the molecular mass, the mixture was analysed by MALDI-TOF MS in reflectron positive ion mode. The instrument was externally calibrated using a tryptic alcohol dehydrogenase digest (Waters, Milford, MA, USA) as standard. A mass accuracy near to the nominal (50 ppm) was achieved for each standard, revealing ion peaks [M+H]^+^ showed in Table 1. The purity was ascertained by RP-HPLC, using Ascentis C18 reverse phase column (5 µm, 4.6 mm × 250 mm) and following gradient elution with A: 0.1% TFA/water, B: 0.1% TFA/ACN; from 0% to 60% of B over 40 min, a flow rate: 1.0 mL/min; detection: UV, 260 and 280 nm. 

### 3.2. NMR Characterization of CA-PNA Dimer 1

1D NMR spectra were recorded for at 300.03 MHz ^1^H on a Varian Mercury Plus 300 Fourier Transform instrument in D_2_O at 25 °C. Temperature and 2D spectra were recorded at 500 MHz on a Varian Unity INOVA in a range of temperature between 25 and 70 °C, δ (chemical shift) in ppm, J (coupling constant) in Hz. TOCSY and ROESY experiments were recorded (both in neat D_2_O or H_2_O\D_2_O 90\10) with mixing times of 70 and 150 ms, respectively. Water suppression, when necessary, was achieved utilizing the DPFGSE sequence [41]. The translation diffusion coefficient (Df) was measured using the pulsed field gradient spin-echo (PFGSE) DOSY experiment [42]. 1D ^1^H spectra of dimer 1: ^1^H-NMR (300 MHz, D_2_O) δ: 8.48 (s, 1H, H-2A), 8.20 (s, 1H, H-8A), 8.17 (s, 1H, H-8G), 7.11 (d, 1H, *J* = 15.9 Hz, H-7), 6.84 (d, 1H, *J* = 1.8 Hz, H-2), 6.77 (dd, 1H, *J* = 8.1 and 1.8 Hz, H-6), 6.73 (d, 1H, *J* = 8.1 Hz, H-5), 6.28 (d, 1H, *J* = 15.9 Hz, H-8), 5.17 (s, 1H, H-h), 5.09 (s, 1H, H-g), 4.25–3.85 (ss, 2H, H-f, H-c), 3.38-3.12 (broad ms, 8H, H-e, H-d, H-b, H-a). ^13^C assignments were made from ^1^H-^13^C HSQC spectra: 152.09 (C-2A), 143.60 (C-7), 140.06 (C-8G), 135.30 (C-8A), 120.40 (C-8), 118.86 (C-6), 117.20 (C-2), 116.98 (C-5), 51.20–38.50 (C-a, C-b, C-d, C-e), 50.00–57.50 (C-c C-f), 48.50–47.20 (C-h, C-g).

### 3.3. Conformation Sampling Method

The conformational ensemble was generated using Cyndi software for each of the two molecules with 200 populations and 200 generations. The epsilon values for the four objectives (VDW energy, torsion energy, GD value, and gyration radius) were set as 20 kcal/mol, 5 kcal/mol, 0.2 Å and 0.1 Å, respectively. The maximum iteration for post processing CG minimization was set to 100, and the convergence criterion based on gradient RMS was set to 0.1 kcal·mol-1·Å-1. No initial optimization against the input conformer was applied and the input conformer was discarded from the final conformer ensembles. 

### 3.4. Cytoprotection Assay

HepG2, SK-N-BE(2), and C6 cell lines were seeded in 96-multiwell plates at a density of 2.0 × 10^4^ cells/well. After 24 h of incubation, cells were treated with compound 1 (5.0, 10.0, 25.0, 50.0, and 100.0 μM), and caffeic acid (CA; 2.5, 5.0, 10.0, 25.0, 50.0, 75 and 100.0 μM). At 48 h of incubation, the medium was removed and replaced with fresh medium containing hydrogen peroxide (H_2_O_2_; 500 µM) as oxidant stressor. Cell viability (CV) was assessed by MTT assay, after one hour of exposure to the oxidant.

## 4. Conclusions

In conclusion, PNA dimers containing guanine conjugated to CA were synthesized and characterized. A thorough NMR analysis of dimer **1** demonstrates how in solution these new PNA-CA dimers are mainly characterized by a mixture of conformers in slow exchange. PNA dimers conjugated to CA exhibited different antioxidative, cytotoxic, cytoprotective activities depending on the nature of nucleobase flanking the guanine as well as the position of guanine nucleobase with respect to CA residue. The compound *CA-ga-*NH_2_ (**2**) exhibited major antiradical and lipid peroxidation inhibitory activities compared to *CA-ag-*NH_2_ (**1**) because of the radical trapping ability of guanine close to CA and/or because of the smaller conformational space sampled by **2**. Importantly, *CA-ga-*NH_2_ (**2**) displayed a low cytotoxic activity, that may favour the use of **2** in doses higher than CA and, given the biostability of amide PNA backbone, a longer effect. Finally, protection of cultured cells from a cytotoxic dose of hydrogen peroxide, specially on SK-N-BE(2) was revealed. 

The present studies warrant further investigations which may lead to the discovery of new stable cytoprotective agents of therapeutic value. 

## Supplementary Materials

Supplementary materials can be accessed at: http://www.mdpi.com/1420-3049/18/8/9147/s1.
