# Supplementary material for: Synthesis and Biological Properties of Caffeic Acid-PNA Dimers Containing Guanine
Source: Molecules. 2013 Jul 31;18(8):9147–62. doi: 10.3390/molecules18089147 (PMC6270098; doi:10.3390/molecules18089147)
Supplement: Supplementary file 1 [file molecules-18-09147-s001.pdf]

Supplementary Materials

Figure S1. MALDI-MS spectrum of crude compound **1**.

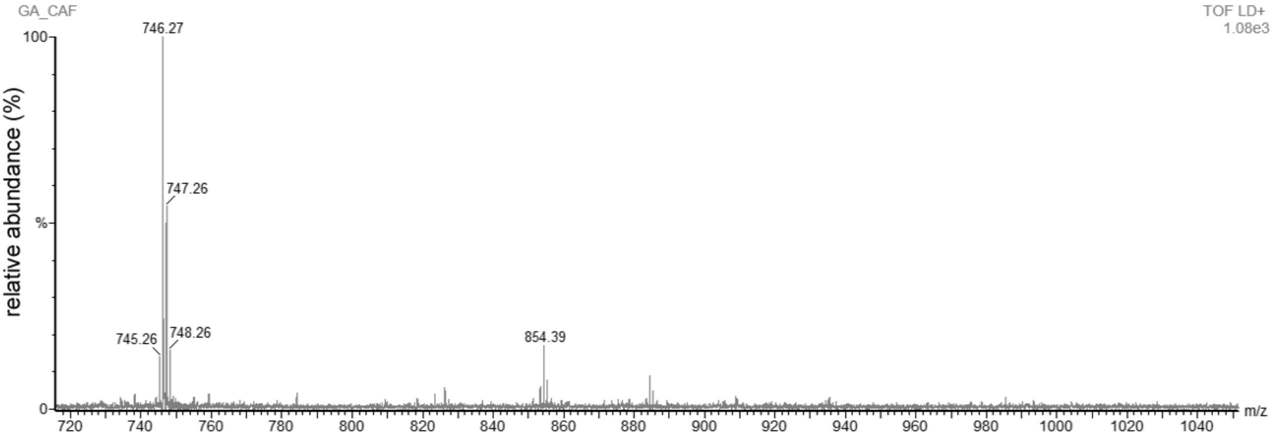

Table S1. DPPH, ABTS and TBARS IC_{50} values of the inhibitor series. IC_{50} values, defined as the compound amount causing 50 per cent activity inhibition, was determined from the plotted curves.

	**DPPH IC_{50}**	**ABTS IC_{50}**	**TBARS IC_{50}**
Caffeic Acid	10.97	2.95	21.79
AG	51.36	13.91	41.38
CG	78.03	13.04	49.0
GA	>100	>100	85.34
GG	>100	36.74	94.36
GC	>100	19.98	47.69
GT	>100	>100	>100
TG	>100	>100	40.92
